# Multi-omics approaches in idiopathic pulmonary fibrosis: from molecular mechanisms to therapeutic targets and precision medicine

**DOI:** 10.3389/fphar.2026.1899849

**Published:** 2026-08-28

**Authors:** Wenbo Hu, Xuehui Wang

**Affiliations:** 1 Heilongjiang University of Chinese Medicine, Harbin, Heilongjiang, China; 2 First Affiliated Hospital of Heilongjiang University of Chinese Medicine, Harbin, Heilongjiang, China

**Keywords:** biomarkers, genomics, idiopathic pulmonary fibrosis, metabolomics, multi-omics, transcriptomics

## Abstract

Idiopathic pulmonary fibrosis (IPF) is a progressive interstitial lung disease with limited therapeutic options and marked molecular heterogeneity. Despite available antifibrotic therapies, disease progression remains poorly predictable, highlighting the need for improved mechanistic understanding and therapeutic targeting. This review summarizes recent advances in multi-omics research to elucidate the molecular mechanisms underlying IPF and to identify potential biomarkers and pharmacological targets. Multi-omics studies, including genomics, epigenomics, transcriptomics, proteomics, metabolomics, microbiome profiling, and single-cell sequencing, have revealed key pathogenic mechanisms in IPF. Genetic susceptibility factors such as *MUC5B* promoter variants and telomere-related genes contribute to disease risk. Epigenetic regulation, including DNA methylation, histone modifications, and non-coding RNAs, plays a central role in fibrotic remodeling. Transcriptomic and proteomic analyses have identified dysregulated signaling pathways, including TGF-β, mTOR, cellular senescence, and extracellular matrix remodeling. Metabolomic alterations indicate disrupted lipid and amino acid metabolism. Importantly, integration of multi-omics datasets enables the identification of molecular endotypes, candidate biomarkers, and potential therapeutic targets. However, challenges including data integration, tissue heterogeneity, limited cohort size, and the need for functional validation remain important barriers to clinical translation. Continued development of multi-omics approaches may facilitate more accurate disease classification and support the development of personalized therapeutic strategies for IPF.

## 1 Introduction

Idiopathic pulmonary fibrosis (IPF) is a rare, chronic, progressive interstitial lung disease characterized by irreversible lung function decline, presenting with cough, chest tightness, wheezing, dyspnea, and potential respiratory failure. The median survival after diagnosis is approximately 3–4 years (Sauleda et al., 2018). Current anti-fibrotic therapies, primarily pirfenidone and nintedanib, are administered uniformly without considering molecular subphenotypes associated with disease progression, and only slow the fibrotic process. Lung transplantation remains the most effective intervention for end-stage IPF, but carries substantial risks, including infection and graft rejection (Spagnolo et al., 2021). Recent advances in multi-omics technologies—genomics, transcriptomics, proteomics, metabolomics, and microbiome—provide powerful tools for elucidating the complex pathophysiology of IPF. Integrating these datasets provides a more comprehensive view of dysregulated molecular networks and offers new opportunities for precise diagnosis and the development of targeted therapeutic strategies.

To ensure the inclusion of the most relevant and high-quality studies, a systematic literature search was conducted using PubMed, Web of Science, and Scopus databases. The keywords are “IPF multi omics,” “IPF biomarkers,” and “IPF pathophysiology.” The focus of the search is on articles published in the past decade, including basic research, translational research, and applied research. The selection criteria include peer-reviewed original research and reviews that provide important insights into the IPF mechanism or demonstrate the application of multi omics techniques. This comprehensive approach aims to critically integrate existing knowledge while identifying gaps and future directions in IPF research.

**FIGURE 1:**
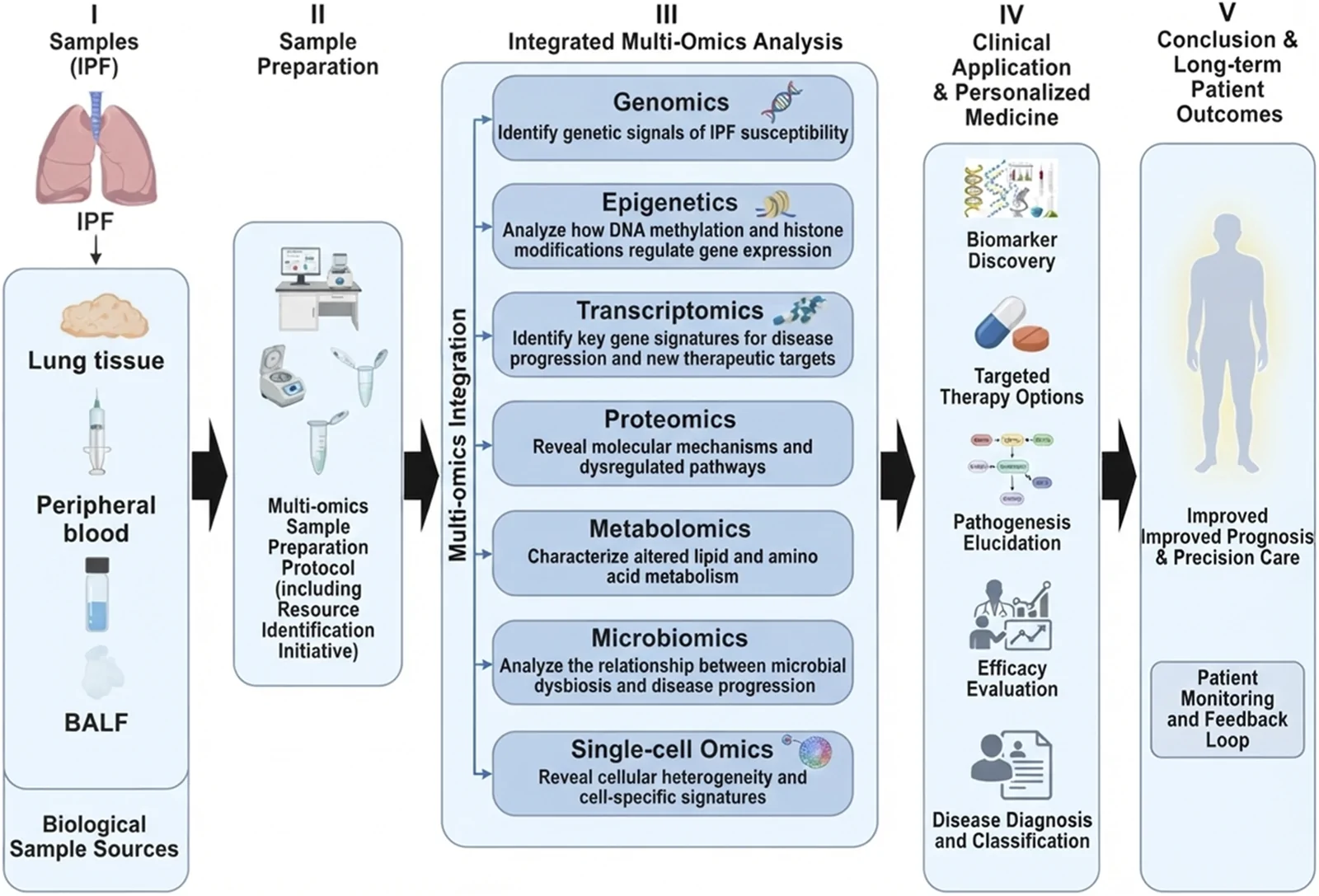
Multi-omics approaches in IPF: From sample analysis to personalized treatment.

This review summarizes how multi-omics technologies have advanced our understanding of IPF, particularly in identifying new biomarkers, clarifying pathogenic mechanisms, and supporting the development of personalized treatment strategies, as shown in Figure 1. By integrating evidence from human clinical samples and experimental models, we aim to provide a balanced perspective that connects basic research with clinical relevance. Overall, this review highlights the transformative potential of multi-omics approaches in addressing key unmet needs in IPF diagnosis and management.

## 2 Pathogenesis and treatment of idiopathic pulmonary fibrosis

Alveolar epithelial cell injury is considered an early pathological event of IPF, followed by fibroblast proliferation and cytokine-induced activation into myofibroblasts, leading to excessive deposition of extracellular matrix (ECM) deposition (Koudstaal et al., 2023). Although inflammation and immune cells contribute to disease progression and fibrosis, IPF is not considered a primarily inflammation-driven disease, unlike some other fibrotic interstitial lung diseases. However, the pathogenesis of IPF remains only partially understood. It involves multiple cell types—alveolar epithelial cells, fibroblasts, macrophages, and immune cells (Hult et al., 2022; Cheng et al., 2025; Yan et al., 2024; Simon et al., 2021)—and several signaling pathways, including transforming growth factor-β (TGF-β), Wnt/β-catenin, mammalian target of rapamycin (mTOR), reactive oxygen species (ROS), and others (Kou et al., 2019; Liu et al., 2021; Platé et al., 2020; Hong et al., 2023; Liu J. et al., 2023), all of which regulate cellular stress responses and chronic inflammation. Emerging evidence indicates that non-coding RNAs, particularly miRNAs, play crucial roles in IPF, serving as diagnostic and prognostic biomarkers (Cho et al., 2020) and influencing the fibrotic microenvironment (Zhang et al., 2025). Further work is needed to clarify these mechanisms and validate additional potential drivers of IPF pathogenesis.

Current IPF management includes both pharmacological and non-pharmacological approaches. The pharmacological management of IPF mainly relies on the anti-fibrotic drugs pirfenidone and nintedanib, which slow disease progression but do not prevent or reverse fibrosis (Bonella et al., 2023). Although corticosteroids and immunosuppressants were used historically, they are no longer recommended for the routine treatment of IPF due to limited benefit and increased risk of adverse outcomes (Arai et al., 2017). However, because of irreversible structural changes in the lung, these therapies often provide limited benefit and are associated with significant side effects. Non-pharmacological options such as oxygen therapy and pulmonary rehabilitation may improve quality of life (Tuyls et al., 2019), though their impact on dyspnea relief and survival remains uncertain (Yang et al., 2025). Lung transplantation can improve symptoms and extend survival, but carries high risks, including perioperative mortality and comorbidities such as cardiac dysfunction, gastroesophageal reflux, and diabetes (Badnes-Bonet et al., 2021). As a result, IPF remains difficult to treat and cure. New strategies are being investigated. Stem cell transplantation and nanotechnology-based drug delivery systems (Ferguson et al., 2023) have shown promise in preclinical studies. For example, Zhao et al. reported that human airway basal cell transplantation in mice generated functional airway and alveolar-like structures and improved fibrosis and survival (Zhao et al., 2025a). Luara et al. showed that inhaled lipid nanoparticles carrying nintedanib increased lung drug concentration by 8000-fold and prolonged its half-life tenfold. Other emerging approaches include miRNA-based therapies and exosome-mediated delivery. Further investigation into the molecular mechanisms of IPF is essential for developing more effective, targeted, and personalized treatments.

## 3 Lessons from other diseases

Omics and systems biology have reshaped the understanding of several diseases, providing a blueprint for advancing IPF study.

Cancer: Integrated multi-omics studies in cancers such as breast and lung cancer have identified driver mutations, immune checkpoint targets, and predictive biomarkers for treatment response. For example, genomic analysis identifying HER2 amplification led to the development of trastuzumab, transforming breast cancer therapy (Tomassetti and Vogelstein, 2017). This underscores how genomics can similarly identify molecular targets in IPF.

Rheumatoid Arthritis (RA): Transcriptomic profiling in RA has mapped key inflammatory cytokine networks, including TNF-α and IL-6, which informed the development of anti-TNF therapies (Orange et al., 2020). These findings suggest that transcriptomic profiling in IPF could identify analogous cytokine targets for anti-inflammatory therapy.

Diabetes: Metabolomics has revealed abnormalities in glucose and lipid metabolism in diabetes (Newgard, 2017; Ludwig and Ebbeling, 2018). Applying similar metabolomic strategies to IPF could help identify metabol ic pathways involved in extracellular matrix deposition and altered energy metabolism.

## 4 Sample preparation for multi-omics studies in IPF

### 4.1 Resource identification initiative

Multi-omics studies in IPF typically use lung tissue, bronchoalveolar lavage fluid (BALF), and peripheral blood, with blood samples being the most common because they are easy to obtain. Transcriptomics, metabolomics, lipidomics, and microbiome analyses are highly sensitive to sample quality and require good purity, adequate concentration, and minimal contamination. Proteomics is generally more tolerant and can be performed on samples that meet basic quality standards.

Advanced approaches such as single-cell omics and spatial transcriptomics are still limited in IPF research. These methods require higher-quality samples but offer the potential for much more detailed information, especially regarding BALF composition and cell-type–specific changes. Their use could greatly improve the analysis of molecular pathways relevant to diagnosis, prognosis, and treatment, helping address current challenges in personalized therapy and biomarkers discovery.

Integrating multi-omics data not only improves our understanding of IPF pathogenesis but also supports patient stratification based on molecular endotypes. It also promotes the development of new anti-fibrotic therapies targeting pathways such as integrins, phosphodiesterase-4 (PDE4), and Hedgehog signaling. Emerging tools like spatial omics and radiomics further link molecular findings with histopathology and clinical features, creating new opportunities for earlier diagnosis, more accurate prognosis, and personalized treatment of IPF.

## 5 Advances in multi-omics research in IPF

### 5.1 Genomics

Genomics, emerging from the Human Genome Project in the late 1980s, focuses on the comprehensive analysis and comparison of genomes to understand their structure, function, evolution, and regulation (Collins et al., 2003). Sequencing genomic DNA is only a starting point; the distribution of nucleotides along chromosomes and how they influence biological processes must also be examined. Beyond protein-coding genes, genomics includes non-coding RNAs, promoters, regulatory elements, and repetitive sequences. The field now encompasses several branches—such as functional genomics, structural genomics, epigenomics, and pharmacogenomics—each offering different perspectives on genome biology. Recent advances in high-throughput sequencing technologies. In IPF, genomic approaches have the potential to clarify genetic risk factors, improve treatment selection, reduce side effects, and ultimately enhance therapeutic outcomes.

In IPF, GWAS have identified nearly 20 independent genetic signals linked to disease risk. These variants are involved in pathways related to telomere maintenance, cell–cell adhesion, host defense, TGF-β signaling, and mitotic spindle assembly (Alle et al., 2022; Partanen et al., 2022; Allen et al., 2017; Allen et al., 2020).

The MUC5B gene encodes a mucin essential for airway defense and is expressed in mucus glands of the terminal and conducting airways, in alveolar type II epithelial cells (AECII), and in epithelial cells of alveolar cysts (Kurches et al., 2024). The promoter variant rs35705950 (T/G) is the strongest known genetic risk factor for IPF (Seibold et al., 2011). Current theories suggest that excess MUC5B reduces mucociliary clearance, lowering the lung’s ability to remove inhaled particles and microbes. These mechanisms are important but do not fully explain disease development, and the exact role of MUC5B in IPF is still not fully defined. Recent multi-omics analyses have also identified MUC1, which encodes a transmembrane mucin, as a new susceptibility locus. In addition, the rs74614704 variant in NPRL3, a regulator of mTORC1 signaling, links mucin-associated pathways to fibrotic remodeling.

DSP (desmoplakin protein) is a core desmosomal protein that helps maintain cell–cell adhesion and tissue integrity. Variants in DSP, including rs2076295, can weaken alveolar epithelial structure, promoting abnormal ECM deposition and accelerating fibrosis (Donoghue et al., 2023). Genetic variants in both MUC5B and DSP also correlate with DNA methylation changes (Hao et al., 2020), suggesting that multiple genetic and epigenetic factors may act together to influence IPF development and progression.

Telomeres are nucleoprotein structures that protect chromosome ends and naturally shorten with each cell division. When telomeres become critically short, they trigger DNA damage response (DDR) signaling. In IPF, sustained DDR activation can impair alveolar epithelial cell (AEC) function and promote cellular senescence (Borie et al., 2022). Mutations in TERT, TERC, PARN, and RTEL1 disrupt telomere maintenance and are linked to the development of pulmonary fibrosis (McDonough et al., 2018). In a longitudinal study of 143 patients, more than half had short telomeres, which were associated with faster lung function decline and a higher frequency of telomere-related gene mutations (Moore et al., 2019).

In IPF, a newly identified NHP2 mutation (p.Y24N) disrupts the nuclear import of the NHP2 protein, lowering the levels of proteins required for telomere stability and telomerase activity (Liu et al., 2013). Telomere dysfunction impairs AECII progenitor cells, limiting their differentiation and regenerative capacity, which contributes to alveolar collapse and fibrosis (Liu L. et al., 2023). GWAS findings now broaden telomere-related risk beyond core telomerase genes. The SPDL1 variant rs116483731, which encodes a spindle assembly protein, has been identified as a new risk factor for accelerated telomere shortening and cellular senescence—likely through mitotic errors that increase replicative stress. Notably, rare functional mutations in TERT, PARN, TERC, and RTEL1 are enriched in IPF patients who do not carry the common MUC5B rs35705950 risk allele, suggesting possible genetic interactions between mucin pathways and telomerase-related mechanisms.

Pulmonary surfactant, a lipid-protein complex synthesized by AECII, maintains alveolar integrity by reducing surface tension. Surfactant dysfunction directly contributes to IPF pathogenesis via AECII injury and aberrant repair (Dressen et al., 2018), leading to decreased alveolar stability, collapse, and fibrosis. Beyond structural roles, surfactant proteins regulate host defense by modulating pro-inflammatory cytokines, chemokines, and tissue repair processes.

Four surfactant-related genes—SFTPA1, SFTPA2, SFTPC, and ABCA3—are associated with familial and sporadic pulmonary fibrosis (as shown in Table 1) (Sutton et al., 2022). Distinct functional roles of SFTPA1 and SFTPA2 may arise from altered expression ratios regulating surfactant activity (Legendre et al., 2020). Autosomal dominant SFTPC mutations, such as SFTPC-i73T, disrupt lamellar body maturation in AECII, causing surfactant accumulation and oxidative stress (Floros et al., 2021). Recessive ABCA3 variants confined to lamellar body membranes are often associated with childhood interstitial lung disease, underscoring surfactant dysregulation as an age-related mechanism.

**TABLE 1:** Gene variants associated with pulmonary fibrosis and their functional impact.

Gene	Variant	Chr	Impact on IPF	References
MUC5B	rs35705950	11	Strongest genetic risk factor; promoter hypomethylation increases mucin production, impairing mucosal defense. Associated with higher CT fibrosis scores	Seibold et al. (2011)
SLC6A6	rs112271207	3	Its role as a taurine transporter suggests epigenetic regulation of IPF pathogenesis	Chin et al. (2025)
NPRL3	rs74614704	16	Regulates mTORCl signaling, linking mucin pathways (e.g., MUC5B) to fibrotic remodeling, May modulate cellular energy metabolism and fibrotic signaling cascades	Alle et al. (2022)
DSP	rs2076295	6	Disrupts alveolar epithelial integrity, accelerating fibrosis, correlates with differential responses to nintedanib therapy	Hao et al. (2020)
TERT	rs4449583	5	Telomere shortening induces alveolar epithelial senescence. Linked to earlyonset IPF, hematologic complications post-transplant,and reduced survival	Peljto et al. (2023)
TERC	rs2293607	3	—	Moore et al. (2019)
PARN	—	—	—	Dressen et al. (2018)
RTEL1	rs41308092	20	—	Peljto et al. (2023)
SPDL1	rs116483731	5	Risk factor accelerating telomere attrition through mitotic errors	Dhindsa et al. (2021)
KNL1	rs12912339	15	Mitotic spindle assembly protein variant implicated in mitotic errors and replicative stress, contributes to alveolar stem cell dysfunction and cellular senescence	Alle et al. (2022)
STMN3	rs112087793	20	Variant associated with cytoskeletal reorganization *via* spindle assembly defects, promotes mitotic stress, impairing alveolar epithelial repair and regeneration	Alle et al. (2022)
SFTPA1	rs1215316727	—	Mutations cause surfactant dysfunction, leading to alveolar collapse and fibrosis, Associated with atypical radiological patterns and rapid lung function decline	Floros et al. (2021)
SFTPC	—	—	—	Dickens et al. (2022)
ABCA3	—	—	—	Sutton et al. (2022)
AKAP13	rs62025270	15	RhoA regulator exacerbates TGF-β dysregulation, increasing fibrotic remodeling	Dressen et al. (2018)
TOLLIP	rs3750920	11	Modifies therapeutie response to N-acetylcysteine; T'T genotype benefits while CC genotype may worsen outcomes	Oldham et al. (2015)
PCSK6	rs35647788	15	Associated with reduced transplant-free survival *via* dysregulated proteolytic processing of profibrotic mediators	Oldham et al. (2023)
PKN2	rs115982800	1	Correlates with rapid FVC decline; regulates cytoskeletal remodeling and fibroblast activation	Allen et al. (2023)

The TGF-β pathway is a core driver of IPF, shaped by both genetic susceptibility and downstream effector mechanisms. TGF-β1 promotes fibroblast-to-myofibroblast differentiation through Smad2/3 activation (Dickens et al., 2022), a process tightly controlled by epigenetic regulation. It also alters histone marks (H3K9me2/3 and H3K4me1/2/3) on promoters of Col1A1, CTGF, and PAI-1, increasing transcription of profibrotic genes (Wang et al., 2022). After moving into the nucleus, Smad complexes function as transcription factors and recruit histone deacetylases to remodel chromatin and further enhance ECM gene expression (Sun et al., 2010).

Wnt signaling intersects with TGF-β at multiple points. Canonical Wnt/β-catenin activation in alveolar epithelium enhances IL-1β-driven amplification of TGF-β (Herrera et al., 2018), whereas non-canonical ligands such as WNT5A drive cytoskeletal changes through JNK and ROCK pathways. Mechanical tension generated by F-actin enables integrin αv-mediated activation of latent TGF-β (Könighoff et al., 2008). Genetic variation in AKAP13, a RhoA regulator, further disrupts TGF-β control and increases IPF risk (Trinh-Minh et al., 2024).

mTOR signaling adds another layer of control. TGF-β activates mTOR through Smad-dependent, PI3K/AKT-independent mechanisms (Liang et al., 2014), and genetic variation within mTOR-related pathways may further modify IPF risk. Together, these interactions highlight how multiple signaling networks converge on TGF-β and underscore the need for therapies that target this broader regulatory landscape. These genetic findings also provide a basis for understanding how epigenetic regulation, gene expression, and protein signaling further shape disease progression, which are discussed in the following sections.

### 5.2 Epigenetics

Epigenetics refers to heritable changes in gene expression that occur without altering the DNA sequence. These include DNA methylation, histone modifications, chromatin remodeling, and regulation by non-coding RNAs, all of which influence transcription or translation (Cavalli and Heard, 2019). While genetic variants contribute to IPF susceptibility, epigenetic mechanisms regulate how these genetic factors are expressed in response to aging, environmental exposure, and tissue injury, providing an important link between inherited risk and disease progression. Advances in next-generation sequencing (NGS) have accelerated epigenetic research and enabled techniques such as whole-genome bisulfite sequencing (WGBS), chromatin immunoprecipitation sequencing (ChIP-seq), and RNA sequencing (RNA-seq). Among these, DNA methylation profiling has become a key approach for dissecting epigenetic mechanisms in human disease.

The rs35705950 risk allele is linked to hypomethylation of the MUC5B promoter, which increases MUC5B transcription. Epigenome-wide studies confirm the importance of DNA methylation in IPF and highlight additional loci such as SLC6A6 rs112271207, a taurine transporter variant with methylation effects. In patient-derived fibroblasts, the CDKN2B locus is hypermethylated, lowering CDKN2B expression and promoting myofibroblast differentiation through SRF and MRTF-A signaling (Scruggs et al., 2018). Experimental models further support a functional role for methylation changes: loss of DNMT3A or overexpression of SOD2 reduces fibrosis in mice, suggesting an “epigenetic–metabolic–fibrotic” axis and pointing to DNMT3A inhibitors or SOD2 agonists as potential therapeutic options (Wang X. C. et al., 2025).

Histones, core components of nucleosomes, play crucial roles in gene expression regulation through post-translational modifications. Early studies showed that the histone deacetylase (HDAC) inhibitor SAHA induces myofibroblast apoptosis in IPF by upregulating pro-apoptotic Bak and downregulating Bcl-xL, accompanied by changes in histone acetylation at regulatory regions (Sanders et al., 2014).

Non-coding RNAs (ncRNAs) regulate gene expression without coding for proteins. Among them, miRNAs are key modulators of fibroblast-to-myofibroblast transition (FMT) and epithelial–mesenchymal transition (EMT). MiR-26a is another epigenetically relevant miRNA with antifibrotic activity in experimental pulmonary fibrosis. Reduced miR-26a expression has been associated with increased TGF-β signaling, fibroblast activation, and ECM accumulation, whereas restoration of miR-26a expression can attenuate fibrotic responses. These findings suggest that altered miRNA networks may contribute to IPF progression by regulating key profibrotic pathways. However, challenges including delivery efficiency, cell-type specificity, off-target effects, and long-term safety need to be addressed before miRNA-based therapies can be translated into clinical applications (Liang et al., 2014). Yuan et al. (2018) showed that miR-542-5p suppresses FMT in silica-induced fibrosis by targeting integrin α6 (Itga6) and disrupting FAK/PI3K/AKT signaling, which reduces fibroblast migration. Exosomal miRNAs also shape cell-to-cell communication in pulmonary fibrosis. Macrophage-derived exosomal miR-328 promote FMT by targeting FAM13A in fibroblasts (Yao et al., 2019). In contrast, exosomes from bone marrow–derived mesenchymal stem cells carry anti-fibrotic miRNAs—miR-148a-3p—that inhibit collagen production and inflammation by targeting Hsp90b, respectively (Jiang et al., 2023). The let-7 family (let-7a1, let-7f1, let-7d), also known as the let-7afd cluster, plays an AT2 cell–specific role by inhibiting EZH2 and pro-fibrotic transcription factors such as BACH1 and MYC. Loss of let-7 activity leads to H3K27 acetylation of these genes and triggers fibrotic signaling (Seaseock et al., 2025). Long non-coding RNAs (lncRNAs) regulate gene expression through chromatin remodeling and transcriptional interference. Pro-fibrotic lncRNAs, including lncRNA-LOC103691771 and lncRNA-IAPF, enhance FMT through the TGF-β/Smad pathway (Cai et al., 2020; Zhang et al., 2022). Others, such as lncRNA-sirt1-AS, act oppositely; it stabilizes SIRT1 protein and suppresses EMT (Qian et al., 2020). Circular RNAs (circRNAs), which form closed RNA loops, can act as miRNA sponges. The anti-fibrotic circRNA-TADA2A limits FMT by sequestering miR-526b and miR-203, restoring Cav1 and Cav2 expression and reducing myofibroblast activation (Li et al., 2020).

These advances highlight the importance of epigenetic mechanisms in IPF and their potential for guiding biomarkers discovery and new therapeutic strategies. Continued work using human models and clinical samples will be crucial for moving these findings toward clinical use. Many of these epigenetic changes alter gene expression, making transcriptomics a valuable tool for examining their downstream effects in IPF.

### 5.3 Transcriptomics

The central dogma of genetics posits that genetic information flows from DNA to protein via messenger RNA (mRNA) under precise regulation. The transcriptome represents the complete set of RNA transcripts produced by a specific tissue or cell under defined developmental or physiological conditions. Transcriptomics enables global analysis of gene function and structure, providing insights into the molecular mechanisms of biological processes and disease pathogenesis (Zormpas et al., 2023).

Transcriptomic research in IPF highlights several major molecular pathways. The PI3K–AKT–mTOR axis has also emerged as a key driver of myofibroblast differentiation and EMT. Within the epitranscriptome, N6-methyladenosine (m6A) RNA modification has emerged as an important regulatory layer. Loss of the m6A writer METTL14 stabilizes the pro-fibrotic transcript DDIT4 in senescence-associated IPF (Li D. et al., 2025). In contrast, the METTL3–YTHDF2 writer–reader complex suppresses the mTOR inhibitor TSC1, leading to activation of the AKT/mTOR pathway (Liu et al., 2025).

Cell stress and death pathways are also markedly altered in IPF. Gene signatures linked to apoptosis, necroptosis, and ferroptosis show strong prognostic value (Sun et al., 2025). Metabolic shifts and lysosomal defects are additional hallmarks. Non-coding RNAs further shape these responses, especially IncRNA. Transcriptome-wide analyses have identified hundreds of dysregulated lncRNAs in IPF lungs (Ma et al., 2025). Functional studies point to key regulators, including lncABCE1-5, whose loss promotes fibrosis through the KRT14–mTOR/AKT axis (Gao et al., 2025), and the anti-fibrotic lncRNA Airn, induced by Ltbp2 deficiency. Other lncRNAs, such as ROCKI and LINC01215, are upregulated by NF-κB and modulate macrophage activation, underscoring their role in immune–fibrotic crosstalk (Schmerer et al., 2025).

Transcriptomic studies have yielded several clinically meaningful findings. Multiple multi-gene signatures now predict disease progression and survival with good accuracy. These include a 7-gene cell-death score that predicts 3-year survival and a plasma cell–related gene signature (PCRGS) centered on ST6GAL1 (Lin et al., 2025). Individual protein biomarkers have also emerged: serum LTBP2 correlates with FVC decline, and EPDR1 is a strong predictor of mortality. Transcriptomics has also helped identify new therapeutic targets. SPP1 is another promising target, particularly for disrupting epithelial–macrophage crosstalk (Du et al., 2025). Despite progress, research on circular RNAs (circRNAs) in IPF remains sparse, representing one of the largest unexplored areas of the non-coding transcriptome in fibrosis. However, changes in gene expression do not always correspond to protein abundance or activity. Therefore, proteomic analysis provides important complementary information by directly measuring proteins and their functional changes in IPF.

### 5.4 Proteomics

Proteomics is the large-scale study of proteins, including their abundance, composition, post-translational modifications (PTMs), and interactions with other proteins or drugs. By capturing the full protein profile of a sample at a given time, proteomics provides a direct molecular snapshot of the pathophysiological state in IPF. Since the early 2000s, advances in mass spectrometry—from gel-based methods to label-free quantification, isotope-labeling approaches (iTRAQ/TMT), and data-independent acquisition (DIA)—have greatly expanded the depth and accuracy of proteomic analysis in IPF research.

Proteomics has expanded our understanding of IPF pathogenesis, moving beyond classical ECM remodeling to reveal roles for immune dysregulation and metabolic reprogramming. An iTRAQ-based analysis of IPF lung tissue characterized the matrisome and confirmed activation of known pathways such as PI3K–Akt and focal adhesion. It also validated several previously reported upregulated proteins in IPF—SCGB1A1, TAGLN, PSEN2, TSPAN1, CTSB, AGR2, SERPINB3, CSPG2, ASPN, HSP90AA1, and HSP90AB1. The study additionally identified new ECM-related proteins, including LGALS7 and ASPN (Tian et al., 2019). In a TLR3 agonist–induced fibrosis model, BALF exosome proteomics demonstrated activation of complement and coagulation pathways, which were suppressed by BRD4 inhibition (Zhao et al., 2019). Another study mapped mitochondrial protein lysine succinylation in IPF lungs, revealing elevated K-succinylation of key metabolic enzymes (such as KYAT3 and IDH2), directly linking mitochondrial dysfunction and post-translational modification changes and supporting the concept of metabolic–epigenetic interaction in IPF (Zhao et al., 2025b).

With advances in spatial proteomics, protein expression can now be mapped directly within intact tissue structures. Using high-throughput spatial mass spectrometry imaging integrated with single-cell sequencing, one study pinpointed COL1A1-high fibroblasts and SPP1-positive macrophages within honeycomb lesions, providing a visual map of how key pathogenic cell populations interact (Maher et al., 2025). Another study applied deep spatial proteomics to characterize honeycomb regions, revealing distinct patterns of matrix deposition and immune cell infiltration (Wuyts et al., 2026).

Proteomics not only reveals new mechanisms but also directly generates numerous novel drug targets. Drug development data analysis indicates that LPAR1 (lysophosphatidic acid receptor 1), ENPP2 (autotaxin, LPA synthase), TGFB1, ROCK1/2 (Rho-associated kinases), and PDE4B are among the most concentrated targets in current pipelines, spanning lipid signaling, classic fibrotic pathways, cytoskeleton, and inflammation. Most of these targets already have drugs in Phase II/III trials, such as the LPAR1 antagonist fipaxalparant and the PDE4B inhibitor nerandomilast. In human lung explant models, proteomics confirmed that the KCa3.1 blocker senicapoc reverses the expression of 118 fibrosis-related proteins, validating its potential as an anti-fibrotic target (Song et al., 2024). BRD4, as an epigenetic regulator, shows that its inhibitor suppresses inflammation and fibrosis in animal models by modulating the exosomal protein profile. Fibroblast activation protein (FAP) is highly expressed on activated fibroblasts in IPF; FAP-targeted peptide-radionuclide conjugates developed based on this are under patent layout for PET imaging and targeted radiotherapy, enabling “theranostics” (Witek et al., 2024).

Proteomics can also be used to evaluate drug responses. In animal models, treatment with a BRD4 inhibitor significantly lowered the levels of proteins such as ORM2, FGA, and FN1 in BALF exosomes, suggesting they could serve as early, local pharmacodynamic markers (Wang F. et al., 2025; Mao et al., 2024; Maxwell et al., 2025). Since proteins regulate many cellular metabolic processes, metabolomics complements proteomics by revealing downstream metabolic changes in IPF.

### 5.5 Metabolomics

Metabolomics examines the types, levels, and changes of endogenous metabolites in living organisms to reveal underlying pathophysiological processes. Because it sits at a central point in the flow of biological information, it can capture signals that reflect genetic, protein, and cellular activity across tissues.

Across multiple studies, metabolic reprogramming in IPF mainly involves lipids, amino acids, and energy metabolism. Among these, abnormal lipid metabolism is the most consistent finding. Numerous reports show marked changes in sphingolipid and phospholipid profiles in the plasma and lung tissue of IPF patients. A large-scale study (n = 300) confirmed elevated sphingolipids such as d43:2 sphingomyelin and increased long-chain acylcarnitines in IPF plasma, pointing to impaired mitochondrial fatty acid β-oxidation (FAO), “fuel” overload, and an ensuing energy deficit (Summer et al., 2024). Spatial metabolomics further revealed pronounced disruption and uneven distribution of phosphatidylcholine (PC) and phosphatidylethanolamine (PE) in fibrotic foci of IPF lungs (Li S. et al., 2025). The marked buildup of long-chain acylcarnitines (e.g., C16-OH/C14-DC) in IPF plasma strongly supports mitochondrial FAO dysfunction. These metabolites also correlate positively with all-cause mortality and act as strong predictors of poor prognosis. In acute exacerbations of IPF (AE-IPF), plasma levels of the arachidonic acid metabolite 5-HETE rise sharply and may serve as an early warning biomarkers (Zhao et al., 2025c). Amino acid metabolism is also disturbed. Abnormal levels of valine, leucine/isoleucine, and other BCAAs in IPF plasma correlate negatively with lung function (e.g., FVC% predicted), possibly reflecting muscle loss and broader metabolic imbalance (Wang F. et al., 2025). In addition, fibroblast activation demands large amounts of proline for collagen production. In IPF, arginine metabolism shifts toward proline synthesis, suggesting disruption of the NO–arginase axis, a potential metabolic switch that promotes fibrosis (Liu et al., 2024).

Mitochondrial dysfunction is a core feature of IPF pathogenesis. The buildup of long-chain acylcarnitines is a clear marker of impaired mitochondrial FAO (Summer et al., 2024). As energy metabolism shifts, fibroblasts and other cells increasingly rely on glycolysis (the Warburg effect), a metabolic state that supports rapid growth and resistance to apoptosis, ultimately promoting persistent fibrotic progression.

Metabolomics is increasingly being used to assess and predict responses to anti-fibrotic therapy. In a longitudinal study of patients treated with nintedanib, higher baseline plasma levels of pPE 18:0p/22:6 were associated with poorer outcomes, suggesting that these patients may benefit from more intensive management or early combination therapy (Shimuzu et al., 2025). This highlights the potential of metabolomics to guide personalized treatment in IPF. Strategies aimed at restoring mitochondrial FAO may help slow fibrosis by correcting energy metabolism disturbances. Likewise, agents that modulate the arginine–proline metabolic axis, including arginase inhibitors, show promise by limiting the supply of precursors needed for collagen production (Liu et al., 2024). Because metabolic pathways are also influenced by host–microbiome interactions, microbiome studies provide additional insight into disease mechanisms and potential therapeutic strategies in IPF.

### 5.6 Microbiomics

The microbiome refers to the collective community of microorganisms and their genetic material within a specific environment or ecosystem, harboring vast microbial resources. The lower respiratory tract in healthy individuals is not sterile; rather, it contains a small, stable community of bacteria. In IPF, this balance is markedly disrupted. Over the past decade, multiple studies have shown that lung bacterial load is significantly higher in IPF patients than in healthy controls. A recent study further linked increased bacterial load to disease progression and mortality, suggesting that “pulmonary microecological imbalance” may represent a potential treatable trait (Puiu et al., 2024). Changes in microbial community structure are also evident. IPF patients show reduced alpha diversity (e.g., lower Shannon and Chao1 indices) and altered beta diversity. Reduced alpha diversity reflects a less rich and less stable microbial community, and patients with progressive fibrotic ILD consistently show lower Shannon indices. Beta diversity analyses reveal a clear shift in microbial composition compared with healthy individuals, indicating a fundamental restructuring of the lung micro-ecosystem (Puiu et al., 2024). Several bacterial genera display abnormal enrichment in BALF or lung tissue from IPF patients, most frequently *Streptococcus*, *Veillonella*, *Prevotella*, and *Haemophilus* (Shadid et al., 2024). These organisms typically reside in the oral cavity or upper airway, and their presence in the lower airway may reflect increased micro-aspiration or impaired lung clearance. Importantly, specific microbial signatures correlate directly with clinical outcomes. Higher levels of *Prevotella* and *Haemophilus* predict the risk of acute exacerbation of IPF (AE-IPF) (Shadid et al., 2024), a life-threatening event with very high mortality. In a bleomycin-induced pulmonary fibrosis mouse model, dominant colonization by *Klebsiella* quasipneumoniae worsened fibrosis by inducing ROS-driven mitochondrial autophagy in macrophages, promoting a pro-fibrotic phenotype (Xu et al., 2025). This demonstrates a direct “bacterial infection–host immune response–tissue fibrosis” pathway and points to *Klebsiella* as a potential therapeutic target.

The gut lung axis is an emerging biological concept that refers to the bidirectional communication between the gut and lungs through microorganisms, metabolites, and immune cells. As the largest microbiome and immune organ in the human body, the imbalance of intestinal homeostasis can affect lung health over long distances. A study analyzed 32 patients with IPF and found that compared to healthy controls, the abundance of Enterobacteriaceae bacteria in the gut of patients was significantly increased, while Bifidobacterium, which has anti-inflammatory potential, was significantly reduced (Göktürk et al., 2024). Interestingly, the study also found that the use of anti fibrotic drugs (such as nintedanib or pirfenidone) only had a slight effect on the UCG-004 genus of the Trichomonas family, indicating that existing treatment methods may not fundamentally correct gut microbiota imbalances. But animal models provide strong evidence for the role of the gut lung axis in IPF. A study used FMT technology to transplant the fecal microbiota of fibrotic mice to sterile mice, and found that the latter also exhibited more severe pulmonary fibrosis. On the contrary, transplanting the microbiota of healthy mice can have a relieving effect (Chioma et al., 2022). This directly proves that the gut microbiota itself has the ability to regulate the degree of pulmonary fibrosis. The same study also found that lactobacilli in the gut can alleviate pulmonary fibrosis through the key inflammatory signaling pathway of IL-6/STAT3/IL-17A.

Although MR has provided some clues, the fundamental question of whether microbial dysbiosis is the cause or effect of IPF has not been fully resolved. Dysregulation may simply be the result of diseases leading to decreased immune function and physiological structural changes, such as bronchiectasis. IPF itself is a highly heterogeneous disease, with significant differences in genetic background, environmental exposure, and disease progression rate among different patients. Therefore, there may not be a unified IPF microbiome feature, but rather multiple microbiome subtypes associated with different clinical subtypes. Almost all evidence on the effectiveness of restorative interventions such as probiotics and FMT comes from preclinical models. The biggest bottleneck currently faced is how to safely and effectively translate these findings into a vulnerable population of IPF patients. To better understand how these molecular and microbial changes affect individual cell populations, single-cell and spatial omics provide higher-resolution analysis of cellular composition, interactions, and tissue organization in IPF.

### 5.7 Single-cell omics

From genomics and epigenetics to transcriptomics, proteomics, and metabolomics, cell function is shaped both by direct interactions with neighboring cells and by signals released into the surrounding microenvironment. Single-cell technologies now allow researchers to profile cell types and their physiological or pathological states with unprecedented resolution.

Single-cell studies have reframed IPF as a condition rooted in epithelial injury followed by a profoundly impaired repair response. One of the most significant discoveries is a pathological epithelial population often referred to as aberrant basal cells. These cells express KRT8 and KRT17 but lack the canonical basal cell marker KRT5. Adams et al. (2020) further captured transitional epithelial states with high-resolution mapping, including an “AT2-to-KRT17” trajectory marked by persistent epigenetic “damage memory.” Another important epithelial subset is the injury-activated AT2 progenitor (IAAP), a PD-L1–positive population with low Sftpc expression. A related study demonstrated that metformin expands this regenerative progenitor pool through the AMPK–FGFR2b signaling axis, directly linking a potential therapeutic agent to a defined reparative mechanism (Lv et al., 2025).

Spatial transcriptomics (ST) has become essential for delineating how diverse cell states assemble into pathological microenvironments. By overlaying gene expression onto intact tissue architecture, ST identifies localized “hotspots” of disease activity, such as fibroblastic foci. Correlative analyses have shown that the PAK1/PAK4 regulon is specifically enriched in mesenchymal cells within these foci, providing a spatially grounded rationale for evaluating PAK inhibition (Watanabe et al., 2025). ST also offers *in situ* validation of cell–cell communication networks inferred from scRNA-seq. For example, it visualizes the co-localization of SPP1^+^ macrophages with activated fibroblasts and maps chemokine gradients that coordinate immune-cell aggregation around honeycomb cysts (Vannan et al., 2025). Together, these spatially resolved insights bridge molecular phenotypes with histopathology, confirming that single-cell–defined populations are not dissociation artifacts but are organized into coherent, disease-driving cellular communities.

Single-cell and spatial studies increasingly support the view that IPF is a spatially organized multicellular ecosystem rather than a homogeneous fibrotic process (Habermann et al., 2020; Mayr et al., 2024). Recurrent epithelial injury and defective regeneration generate abnormal transitional epithelial states, including KRT5-negative/KRT17-positive aberrant basaloid cells and other injury-associated alveolar epithelial populations (Kathiriya et al., 2022). These epithelial states coexist with activated fibroblast and myofibroblast populations, SPP1-positive macrophages, endothelial alterations, and localized immune niches (Du et al., 2025; Mayr et al., 2024). Their spatial proximity within fibroblastic foci, honeycomb regions, and fibrotic borders suggests that epithelial dysfunction, macrophage-fibroblast crosstalk, TGF-β signaling, extracellular matrix remodeling, and local immune activation are coordinated within disease-specific microenvironments rather than operating independently (Weeratunga et al., 2025).

These insights also have important implications for pharmacological target validation. A target identified in bulk tissue may reflect changes in cell composition rather than a cell-intrinsic pathogenic mechanism, whereas single-cell and spatial approaches can identify the cell type and tissue niche in which a pathway is active. However, spatial association alone does not establish causality, and therapeutic prioritization requires complementary functional studies using patient-derived cells, precision-cut lung slices, organoids, *ex vivo* lung tissue, and appropriately selected animal models (Lang et al., 2023; Ptasinski et al., 2023).

The bleomycin-induced mouse model remains useful for studying acute injury, inflammatory responses, and early fibrotic mechanisms, but it does not fully reproduce the chronic, age-associated, and largely irreversible nature of human IPF. In particular, bleomycin injury is often followed by relatively active epithelial regeneration and partial fibrosis resolution, whereas human IPF is characterized by persistent epithelial dysfunction, abnormal repair, and progressive architectural remodeling (Brazee et al., 2025). Therefore, findings from bleomycin models should be interpreted as mechanistic or proof-of-concept evidence and should not be directly extrapolated to clinical efficacy without validation in human IPF tissues and human-relevant experimental systems.

The key findings, sample types, representative studies, and translational implications of different omics approaches in IPF are summarized in Table 2. Single-cell and spatial omics have transformed our understanding of IPF. They reveal fibrosis not as a uniform process but as a dynamic, complex cellular ecosystem, with a mosaic of abnormal epithelial progenitors, diverse fibroblast populations, and pro-fibrotic immune niches. This detailed mapping improves our mechanistic insight and identifies numerous potential therapeutic targets and biomarkers. Ongoing advances in these technologies are likely to accelerate the development of personalized therapies and usher in a new era of precision medicine for IPF. However, each omics approach provides only part of the disease process. Integrating information across multiple omics layers offers a more comprehensive view of IPF and may improve the identification of disease endotypes, biomarkers, and therapeutic targets.

**TABLE 2:** Key findings and translational value across omics layers in IPF.

Omics level	Common sample types/platforms	Representative findings in IPF	Potential translational value	Major limitations
Genomics	Blood DNA, lung tissue; GWAS, WGS, targeted sequencing	MUC5B promoter variant; telomere-related genes including TERT, TERC, PARN, and RTEL1; variants associated with epithelial integrity and host defense	Risk stratification, familial pulmonary fibrosis assessment, target prioritization, pharmacogenomic hypotheses	Genetic risk does not directly determine disease onset or treatment response; ancestry representation remains limited (Seibold et al., 2011; Dressen et al., 2018)
Epigenomics	Lung tissue, fibroblasts, epithelial cells; DNA methylation profiling, ChIP-seq, ATAC-seq	Dysregulated DNA methylation, histone modifications, chromatin remodeling, and non-coding RNA networks	Identification of reversible regulatory mechanisms and candidate biomarkers	Cell-type specificity, tissue heterogeneity, and uncertainty regarding causality (Wang et al., 2022; Cavalli and Heard, 2019)
Transcriptomics	Whole lung, blood, BALF, sorted cells; bulk RNA-seq, scRNA-seq	Aberrant epithelial states, fibroblast activation, senescence, TGF-β, mTOR, ER stress, and cell-death programs	Prognostic signatures, molecular endotyping, target discovery	Bulk profiles are confounded by cell composition; signatures require external validation (Platé et al., 2020; Adams et al., 2020)
Proteomics	Plasma, BALF, lung tissue; mass spectrometry, aptamer-based platforms	ECM remodeling, immune mediators, matrisome alterations, and circulating proteins associated with progression	Non-invasive biomarkers, pharmacodynamic markers, target prioritization	Peripheral blood proteins may not reflect target-organ biology; platform coverage is incomplete (Tian et al., 2019; Song et al., 2024)
Metabolomics/lipidomics	Plasma, serum, BALF, lung tissue; LC-MS, GC-MS, spatial metabolomics	Altered sphingolipids, acylcarnitines, phospholipids, amino-acid metabolism, and mitochondrial fatty-acid oxidation	Prognosis, treatment-response hypotheses, metabolic target discovery	Diet, comorbidities, medication use, and pre-analytical factors affect metabolite profiles (Summer et al., 2024; Zhao et al., 2025c)
Microbiomics	BALF, sputum, lung tissue, stool; 16S rRNA sequencing, metagenomics	Altered bacterial burden and composition; potential gut-lung interactions	Identification of treatable traits and host-microbe interactions	Contamination, reverse causation, antibiotic effects, and limited interventional evidence (Puiu et al., 2024; Göktürk et al., 2024)
Single-cell and spatial omics	Fresh/frozen lung tissue; scRNA-seq, spatial transcriptomics, spatial proteomics	Aberrant epithelial states, SPP1-positive macrophages, activated fibroblasts, fibrotic niches, and cell-cell interactions	Cell-type-specific target identification and validation of disease niches	Limited tissue availability, technical cost, dissociation bias, and challenges in longitudinal sampling (Adams et al., 2020)
Integrated multi-omics	Matched blood, BALF, lung tissue, imaging and clinical data	Molecular endotypes, convergent pathways, potential causal targets, and spatially localized disease modules	Trial enrichment, patient stratification, response prediction, and precision pharmacology	Batch effects, sample size, cross-platform harmonization, lack of prospective validation (Maher et al., 2025)

### 5.8 Multi-omics integration strategies and translational applications in IPF

The value of multi-omics research in IPF is not only to generate molecular profiles from different biological levels, but also to integrate these data to identify disease endotypes, regulatory pathways, biomarkers, and potential therapeutic targets. Several approaches have been developed for multi-omics integration. Network-based methods combine information from different omics layers to identify key molecular pathways involved in disease progression. Similarity Network Fusion and clustering approaches integrate patient-level molecular data to classify patients into subgroups with different molecular features and clinical outcomes. Multi-modal factor analysis methods can identify common biological patterns across different datasets. In addition, integrating genetic variants with expression quantitative trait loci (eQTLs), protein quantitative trait loci (pQTLs), colocalization analysis, and Mendelian randomization can help prioritize molecular changes that may contribute to IPF development and progression (Wen et al., 2025; Chen et al., 2024).

Several recent studies have demonstrated the potential of multi-omics integration in IPF. Using samples from the IPF-PRO Registry, Ruan et al. integrated peripheral blood proteomic, total RNA, and miRNA profiles with Similarity Network Fusion and identified two molecular endotypes (Ruan et al., 2023). The endotype associated with more severe disease showed shorter progression-free survival after adjustment for baseline disease severity and antifibrotic treatment. Proteomic and miRNA profiles contributed more to endotype classification than total RNA data. Pathway analysis identified changes in mTOR, VEGF, PDGF, ERK/MAPK, oxidative stress, and immune-related pathways, suggesting that integrated molecular profiles may help identify patient subgroups and guide future clinical studies (Wen et al., 2025; Chen et al., 2024).

Multi-omics approaches can also help prioritize potential therapeutic targets by integrating genetic and molecular evidence. Li et al. combined gene expression, protein abundance, methylation-QTL, genome-wide association, Mendelian randomization, colocalization analysis, bulk transcriptomics, and single-cell data to identify mitochondria-related genes associated with IPF susceptibility (Li X. et al., 2025). Their study identified several candidate genes, including POLG, NDUFB10, NBR1, MCL1, and MTX1, with some genes showing enrichment in myofibroblasts. These findings highlight the potential role of mitochondrial pathways in IPF and provide candidates for further investigation. However, functional validation using human-relevant models is still needed before these targets can be considered for therapeutic development.

Spatial information is important for understanding IPF because fibrotic changes occur in specific regions of the lung rather than being evenly distributed. Franzén et al. used spatial transcriptomics to map human IPF and bleomycin-induced mouse fibrosis and identified fibrotic niches characterized by abnormal alveolar epithelial cells and a TGF-β-enriched microenvironment (Franzén et al., 2024). The study also showed differences between human IPF, where alveolar regeneration is impaired, and the bleomycin model, which shows a more active repair response. These findings highlight the importance of considering tissue location and species differences when evaluating potential therapeutic targets.

Despite the potential of multi-omics approaches for personalized medicine, several challenges remain before their clinical application. Multi-omics datasets can be affected by batch effects, platform differences, variable sample quality, tissue heterogeneity, limited sample sizes, and the lack of longitudinal clinical data. In addition, molecular signatures identified in blood or other accessible samples may not fully represent disease processes occurring in the lung. Although approaches such as Mendelian randomization can provide evidence for potential causal relationships, functional validation is still needed to confirm disease mechanisms and therapeutic targets. Future studies using independent and diverse patient cohorts, together with prospective clinical trials, are needed to determine whether multi-omics-based patient stratification can improve treatment selection and clinical outcomes. Several multi-omics-informed therapeutic targets and precision pharmacology strategies currently under investigation are summarized in Table 3.

**TABLE 3:** Multi-omics-informed therapeutic targets and precision pharmacology strategies in idiopathic pulmonary fibrosis.

Pathway/target	Therapeutic strategy or representative intervention	Multi-omics rationale	Current development stage	Potential patient subgroup	Key limitations
Multiple profibrotic receptor tyrosine kinases	Nintedanib	Transcriptomic and proteomic data support PDGF, FGF, VEGF, and fibroblast activation pathways	Approved antifibrotic therapy	Broad IPF population; no validated omics-defined responder group	Slows progression but does not reverse fibrosis; gastrointestinal and hepatic adverse effects (Lancaster et al., 2019)
Broad antifibrotic/oxidative and cytokine-related pathways	Pirfenidone	Multi-pathway antifibrotic activity; may affect TGF-β-related signaling and oxidative stress	Approved antifibrotic therapy	Broad IPF population; no validated molecular selection strategy	Modest effect size; photosensitivity and gastrointestinal intolerance (Maher et al., 2019)
Integrin-mediated activation of latent TGF-β	αvβ6/αv integrin inhibition, e.g., bexotegrast and related strategies	Spatial and transcriptomic studies show localized TGF-β activity in fibrotic niches; integrin expression is enriched in injured epithelium and fibrotic remodeling	Clinical development	Patients with high epithelial injury/TGF-β activation signatures	Safety, target specificity, optimal biomarker thresholds, and clinical validation remain unresolved (Spagnolo et al., 2021)
PDE4B signaling	Selective PDE4B inhibition, e.g., nerandomilast	Multi-omics studies implicate inflammatory-fibrotic signaling, fibroblast activation, and immune-stromal crosstalk	Late-stage clinical development	Potentially patients with PDE4B-associated inflammatory-fibrotic signatures	Biomarker-guided selection has not been established; tolerability must be considered (Bonella et al., 2023)
Lysophosphatidic acid pathway	LPA/LPAR1 pathway inhibition, e.g., fipaxalparant	Lipidomic and proteomic evidence supports altered lipid signaling and fibroblast activation	Clinical development	Patients with lipid-remodeling or LPA-associated signatures	Previous failures in the pathway highlight uncertainty in target engagement and patient selection (Spagnolo et al., 2021; Bonella et al., 2023)
Mitochondrial dysfunction and metabolic reprogramming	Restoration of mitochondrial homeostasis, fatty-acid oxidation, redox balance, or metabolic pathway modulation	Metabolomics and causal-inference studies implicate acylcarnitines, mitochondrial dysfunction, and mitochondria-related genes	Preclinical/early translational	Patients with mitochondrial or metabolic-risk signatures	Causal mechanisms, safety, and clinically actionable biomarkers remain uncertain (Wang X. C. et al., 2025)
Senescence and defective epithelial regeneration	Senolytic, regenerative, AMPK-related, or epithelial repair strategies	Genomic, transcriptomic, and spatial data support epithelial senescence and failed AT2-to-AT1 differentiation	Preclinical to early clinical investigation	Patients with telomere dysfunction, epithelial senescence, or abnormal transitional epithelial-cell signatures	Risk of impairing physiological repair; limited human efficacy data (Zhao et al., 2025a; Seaseock et al., 2025)
SPP1-positive macrophage-fibroblast crosstalk	Macrophage reprogramming, SPP1-related or chemokine-pathway interventions	Single-cell and spatial studies identify macrophage-fibroblast proximity in fibrotic niches	Preclinical	Patients with macrophage-rich, immune-fibrotic endotypes	Macrophage heterogeneity and potential host-defense impairment (Du et al., 2025)
Non-coding RNA and extracellular-vesicle approaches	miRNA mimics/inhibitors, engineered extracellular vesicles	Epigenomic and transcriptomic studies identify dysregulated miRNA networks including miR-26a and miR-21	Preclinical	Molecularly selected patients after robust validation	Delivery, cell specificity, off-target effects, durability, and manufacturing challenges (Liang et al., 2014; Yao et al., 2019)
Microbiome-directed interventions	Antibiotic stewardship, microbiome modulation, probiotic or FMT-related strategies	Microbiome studies suggest altered bacterial burden and gut-lung interactions	Observational/preclinical	Patients with reproducible microbial endotypes, if validated	Causality is unresolved; no established clinical microbiome-directed therapy for IPF (Xu et al., 2025)

## 6 Conclusion

This review summarizes the pathogenesis and current treatment strategies for IPF, highlighting the key role of multi-omics technologies in unraveling its complex molecular networks. Current management, relying mainly on anti-fibrotic drugs and lung transplantation, has limited efficacy and lacks personalized approaches. We provide a comprehensive overview of how multi-omics can be applied in IPF research, from sample preparation and technical implementation to experimental discovery and clinical translation. We encourage clinicians to adopt a multi-omics perspective to tackle the high heterogeneity and poor prognosis of IPF, enabling more precise, stratified, and personalized treatment strategies. By integrating data from genomics, epigenetics, transcriptomics, proteomics, metabolomics, and the microbiome, clinicians may identify diagnostic and prognostic biomarkers in blood, BALF, or lung tissue, supporting better-targeted therapies, improved responses, and prolonged survival.

Multi-omics integration is reshaping our understanding of IPF, moving from traditional pathological descriptions to mechanism-based classification using molecular endotypes. Genomics has identified key susceptibility genes, including MUC5B, TERT, and DSP, and revealed regulatory networks involving telomere maintenance, mucin secretion, and mTOR signaling. Epigenetic studies have highlighted how gene-environment interactions drive fibroblast activation and epithelial senescence through DNA methylation, histone modifications, and non-coding RNAs. Transcriptomics and proteomics have confirmed classic pathways like TGF-β and PI3K-AKT while uncovering new pathogenic modules linked to cell death programs, m6A modification, and mitochondrial metabolism. Metabolomics shows that dysregulated lipid metabolism and impaired fatty acid oxidation contribute to the IPF energy crisis. Microbiomics demonstrates that lung and gut microbial imbalances influence disease progression, opening new avenues for studying the “microbiota–immune–fibrosis” axis.

Multi-omics integration offers a comprehensive perspective for IPF research. By combining data across different omics layers, researchers can build disease-specific molecular networks and uncover regulatory relationships across biological systems. Inspired by the success of multi-omics in chronic diseases such as cancer and rheumatoid arthritis, this review highlights its transformative potential in IPF—enhancing mechanistic understanding while enabling the discovery of novel biomarkers and precision therapies. Future efforts should focus on: (1) advancing single-cell and spatial multi-omics to clarify cell-type specificity and the role of tissue architecture in fibrosis; (2) strengthening translational platforms to connect laboratory findings with clinical validation; and (3) promoting collaboration between clinicians and basic scientists to translate multi-omics signatures into clinical subtypes and treatment strategies.

This integrated analysis can help identify key driver biomarkers and establish patient stratification systems based on molecular endotypes, paving the way for truly personalized medicine. Multi-omics-guided patient classification could enable early diagnostics, prognostic assessment, and tailored treatment regimens. Over time, these advances are expected to transform the clinical management of IPF and open new avenues for developing anti-fibrotic drugs and combination therapies.

While many clinically promising biomarkers can be detected in blood or BALF, high-quality human lung tissue remains essential for basic research. Obtaining such tissue is challenging, and autopsy samples often suffer from issues like RNA degradation. Consequently, organoids and genetically engineered animal models remain vital for mechanistic omics studies. Looking ahead, patient-derived iPSCs differentiated into lung epithelial cells or fibroblasts offer promising platforms for human-specific multi-omics research.

In conclusion, integrated multi-omics analysis is guiding IPF research into a new era of systems medicine. With expanding data dimensions, upgraded analytical tools, and deepened clinical collaboration, we are confident that in the near future, earlier detection, more accurate classification, and more personalized treatment for this refractory disease will be achieved, ultimately improving patients’ quality of life and prognosis.
